# Mediating Effect of Financial Behaviour on the Relationship Between Perceived Financial Wellbeing and Its Factors Among Low-Income Young Adults in Malaysia

**DOI:** 10.3389/fpsyg.2022.858630

**Published:** 2022-05-19

**Authors:** Mohamad Fazli Sabri, Rozita Wahab, Nurul Shahnaz Mahdzan, Amirah Shazana Magli, Husniyah Abd Rahim

**Affiliations:** ^1^Department of Resource Management and Consumer Studies, Faculty of Human Ecology, Universiti Putra Malaysia, Serdang, Malaysia; ^2^Department of Human Development and Family Studies, Faculty of Human Ecology, Universiti Putra Malaysia, Serdang, Malaysia; ^3^Department of Finance, Faculty of Business and Economics, University of Malaya, Kuala Lumpur, Malaysia

**Keywords:** financial wellbeing, financial behaviour, financial knowledge, locus of control, young adults, mediating effect

## Abstract

The main objective of this study is to investigate the associations between financial knowledge, locus of control (LOC) and perceived financial wellbeing (FWB) with financial behaviour as a mediator among young adults from low-income households in Malaysia, controlling for education and income. The sample of this study consisted of 482 young adults from five different zones in Peninsular and East Malaysia, which were selected using a multi-stage sampling technique. Data were collected using a set of questionnaire-based surveys. The data were then analysed using Covariance Based Structural Equation Modelling (SEM). The study found that financial knowledge and external LOC as well as financial behaviour were significantly correlated with the perceived FWB of low-income young adults in Malaysia. The results also revealed that financial behaviour mediates the influence by financial knowledge and internal LOC on perceived FWB controlling for education and income. The findings of this study provide insights into the factors of perceived FWB of low-income young adults in Peninsular and East Malaysia. Policymakers, government and non-government organisations may utilise this study to develop new policies, financial programmes or campaigns to enhance the FWB of low-income young adults in Malaysia.

## Introduction

Today, a number of young adults are under threat in terms of their economic and financial wellbeing (hereafter, FWB). Consumerism tempts them to spend more money, whereby online shopping platforms offer a simple way to purchase, and the credit card system provides them with a convenient and transparent means of borrowing money. Credit cards are a necessity for most young adults, including those from the low-income category. Some of them could not even last a month relying with only their monthly income without credit cards (Credit Counselling and Debt Management Agency, 2018).

The issue of FWB is crucial for young adults. Young adults between 18 and 35 years old (Petry, 2002; Shim et al., 2009; Addo, 2017) consist of a few categories. First, young adults could be students or student workers just starting college, needing to manage their expenses and paying for education fees. They could also be unemployed or fresh graduates who are still looking for a decent job or new workers at the companies who usually have the lowest salaries compared to their senior officers. Young adults could be the newlyweds starting their own families or new parents, who are responsible for their families and young children. At this stage of life, while adolescents are still fully supported financially and older adults may be more stable in finance, young adults must start learning how to be financially independent and make most of their financial decisions by themselves (Arnett, 2001). They need money the most at this stage, yet they still have no (or minimum) savings and assets, and they no longer receive full financial support from their parents or family.

In a nationwide study of Americans, the survey reported that 50 percent of young adults had no emergency savings. In terms of financial behaviour, 32 percent of them only afford to pay the credit card bills each month at minimum amount because of their financial constraints. A 63 percent of young adults also failed to answer more than three of the five questions in a financial literacy test, demonstrating that many young adults have insufficient financial knowledge, which indirectly affects their financial decision-making abilities (FINRA Investor Education Foundation, 2015). Based on data from the Asian Institute of Finance (2015), the number of young adults facing bankruptcy in Malaysia is also high. As stated by the Malaysian Department of Insolvency, the number of young adults aged below 35 years declared bankruptcy from 2016 to 2020 as 18,836, equivalent to 25.3 percent of the total bankruptcy cases in Malaysia (Bankruptcy Statistic, 2020). This is indeed a worrying trend among young adults who are at the brink of becoming independent.

Moreover, according to a study conducted by the Consumer Research and Resources Centre (2012), nearly half of young workers aged 18–35 years old were in serious debt, with those earning between RM2,000 and RM3,000 per month having the most debt problems. In addition, based on a survey conducted by the Credit Counselling and Debt Management Agency (2018), almost half of Malaysian working adults fall under ‘need attention’ and ‘under pressure’ categories in terms of their FWB scores. Concerning financial behaviours, two out of 10 working adults reported that they had no savings at all in the previous 6 months. In addition, more than half of those who earn less than RM2,000 cannot afford to save RM1,000 for emergency expenses. Three out of 10 of them also confessed that they sometimes had to borrow money to buy necessities. Therefore, the majority of young adults in Malaysia rely heavily on loans and credit cards. These issues are most likely due to the acquisition of assets such as a house and car, which increased their loan repayment obligations. Given these figures, it is no surprise that young adults have reported lower FWB because of their low financial literacy and poor financial behaviour.

Many crucial financial decisions are made for the first time during young adulthood (Eccles et al., 2013). In order to live financially independent and responsibly, young adults must open a savings account and learn how to use a credit card, which is a fundamental issue in the financial system. Therefore, compared to earlier generations, current young adults appear to have more difficulty making personal financial decisions (Shim et al., 2013). This is partly because they have to face increasingly complex consumer choices in the marketplace. It is also because they are now coming of age amid the uncertain and changing economic realities caused by the financial market collapse, which has created financial distress. Financial behaviour and psychological traits such as LOC developed during this life have broad implications, as they remain throughout adulthood and lay the groundwork for long-term FWB (Xu et al., 2015).

According to Sorgente and Lanz (2017), FWB is a favourable situation that includes both objective and subjective components. The former refers to the material resources that an individual has when the balance between entry (e.g., income) and exit (e.g., debt) is considered, as well as those that the individual already has (e.g., assets, savings accounts and education). Meanwhile, the subjective component refers to the emotional state of a person and the cognitive assessment of their financial situation or subjective experience of it. During the period after high school, most young adults start transiting from financial dependence to independence. Consequently, social scientists believe that possessing financial knowledge is one of the major developmental tasks accomplished during this stage of their lives. The financial knowledge they learn and the financial behaviour or habits they acquire throughout these years, whether positive or negative, will likely influence the decisions they make for their entire lives (Shim et al., 2013). Previous studies have demonstrated the predictive power of locus of control (LOC) for many life outcome variables, including FWB (Conell-Price and Jamison, 2012; Furnham and Cheng, 2017). LOC also influences attitudes and behaviour concerning money (Lim et al., 2003; Furnham and Cheng, 2015). Those with an internal LOC tend to be more strongly motivated to exercise plans and take responsibility for their actions, including finance (Ullah and Yusheng, 2020).

FWB is critical for young adults because it can affect physical, psychological and social health, leading to poor job performance, lack of focus, lower productivity and disengagement in daily routines (Ullah and Yusheng, 2020). Nowadays, young adults face many significant challenges in their FWB. Without exception, Malaysian young adults are also conscious of their financial difficulties due to the rising cost of living. These problems have impacted on how they consume, invest, save and manage challenges to maintain their standard of living in the long run (Sabri et al., 2020b). For instance, they are expected to handle these challenges and achieve FWB, but most fail to do so. Hence, exploring the FWB of young adults in Malaysia is an intriguing and relevant research topic that deserves to be examined from multiple perspectives, as young adults regularly encounter critical financial situations and must make complex financial decisions on their own (Sundarasen and Rahman, 2017). Therefore, the main objective of this paper is to investigate the mediating effect of financial behaviour on the relationship between financial knowledge, internal and external LOC, and FWB among young adults in Malaysia, particularly among low-income households.

Financial behaviour is used as a mediator in this study based on the propositions of the Family Resource Management Theory by Deacon and Firebaugh (1988). According to the theory, family resources consisting of financial resources, financial knowledge and background characteristics of an individual act as inputs entering into a throughput system to produce desired outputs such as achieving one’s financial goals. The throughput system acts as a means in which the inputs undergo a transformation process of financial management including planning, implementing and evaluation of various family financial matters such as managing cash, credit, saving, investments and estate planning (Deacon and Firebaugh, 1988). The theory posits that there are two subsystems in the throughput, denoted as the personal and managerial subsystems. The personal subsystem comprises the psychological aspect, namely, financial attitudes that are shaped through cognitive, emotional and social factors, while the managerial subsystem comprises the financial management aspect in which include planning, evaluating choices and making decisions. Both the subsystems interact with each other while evaluating alternatives in the decision-making process, with the ultimate aim of producing a certain output such as attaining high levels of FWB.

Other studies have also supported the input-throughput-output conceptualisation of the Deacon-Firebaugh model. In a systematic literature review analysis, Goyal et al. (2021) suggest that financial behaviour (termed in their paper as ‘personal financial management behaviour’) is shaped by certain factors such as the socio-demographic background of individuals (e.g., income, education and job status), and psychological factors such as financial attitudes, financial knowledge and locus of control. Subsequently, financial behaviour leads to other outcomes including financial satisfaction, FWB, financial capability and financial security. These ideas suggest that financial behaviour acts as a mediator in which the inputs (demographic characteristics and psychological variables) are transformed into behaviour that contribute towards achieving positive financial outcomes.

Empirical studies have also reinforced the postulation of the mediating effect of financial management or financial behaviour in the determination of FWB. For example, there are numerous studies that provide empirical evidence on the mediating effect of financial behaviour on the relationship between financial knowledge and FWB (e.g., Falahati et al., 2012; Jorgensen et al., 2017; Xiao and Porto, 2017; Saurabh and Nandan, 2018; Setiyani and Solichatun, 2019). These studies advocate that financial knowledge transmutes into specific actions that are implemented to achieve desired financial goals. Meanwhile, studies that use financial behaviour as a mediator between locus of control and financial outcomes are present but relatively scant. One recent study was conducted by She et al. (2022) who examined the mediating role of financial behaviour in the relationship between locus of control and FWB, and found a partial mediating effect of financial behaviour on the aforesaid relationship. The researchers explain that FWB is attained by applying the right behaviour, which are shaped based on the individual’s personal beliefs, value, knowledge and experience. Similarly, Vuković and Pivac (2021) found that financial behaviour significantly mediates the relationship between self-control and financial security. These findings suggest that having control and confidence in actions and attitudes translates into positive actions and behaviour, subsequently leading to higher levels of financial satisfaction.

The present study contributes to the literature by incorporating financial behaviour as a mediator between LOC and perceived FWB, which is scant in the literature. Past studies have usually examined the direct relationship between LOC and financial behaviour (e.g., Britt et al., 2013; Arifin et al., 2018; Parmitasari et al., 2020; Adiputra, 2021; Triono, 2021), the direct relationship between LOC and FWB, wellness or satisfaction (Prawitz and Cohart, 2016; Adiputra, 2021) or the mediating effect of LOC on the relationship between certain antecedents and FWB or financial outcomes (e.g., Grable et al., 2015; Bulmash, 2016; Furnham and Cheng, 2017; Ullah and Yusheng, 2020; Peetz et al., 2021), which is in line with the study by She et al. (2022). However, the present study differs from She et al. (2022) by including two important socio-demographic variables into the model, i.e., income and education. The incorporation of these variables is aligned to the proposition of the Family Resource Management Model suggesting that idiosyncratic socio-demographic characteristics influences personal financial management behaviour.

The remainder of this paper is structured as follows. Section “Literature Review” reviews the relevant literature on FWB and its factors, and presents the hypotheses development. Section “Research Methodology” explains the research methodology undertaken. Section “Findings and Discussion” discusses the findings of the study, and finally, Section “Conclusion and Implications” concludes the paper with a discussion on the implications, limitations and recommendations.

## Literature Review

### Financial Wellbeing

The concept of FWB has been discussed in several academic disciplines, including financial counselling and planning, economics, consumer decision-making and developmental psychology. However, there is no universally accepted measure or definition so far, and there is little clarity on its concept and components. According to Drever et al. (2015), FWB refers to the ability to manage money, deal with financial uncertainties, achieve financial goals and have the financial freedom to make decisions that allow individuals to enjoy their lives. At the same time, the Consumer Financial Protection Bureau (2015) defines FWB as a state of being in which a person can fully meet current and ongoing financial responsibilities, feel safe in their financial future and can make decisions for them to enjoy life. Meanwhile, Shim et al. (2009) briefly explained FWB as the overall satisfaction of individuals with their financial situation.

In terms of their approach, existing definitions and measurements fall into three categories. Some social scientists define FWB using both objective and subjective approaches, while others use only one. For instance, Danes and Yang (2014) explained FWB *via* an objective approach, while Shim et al. (2009) and Joo (2008) used a subjective approach. However, past literature indicates that the subjective approach is more comprehensive and may include non-financial aspects, making it suitable for defining and assessing a complex and personal phenomenon of FWB (Brüggen et al., 2017).

Previous literature suggests that many factors can influence FWB. One of the reasons for financial problems that decrease FWB is the lack of financial knowledge or financial illiteracy experienced by individuals (Sabri and Falahati, 2013; Sabri and Zakaria, 2015). Poor financial behaviours are also regularly discussed as factors that affect FWB (Perry and Morris, 2005; Prawitz and Cohart, 2016; Sabri et al., 2020a). Past studies also sometimes include LOC as a crucial factor associated with financial behaviour and FWB (Grable et al., 2015; Radianto et al., 2021). In addition, a study by Sabri et al. (2020b) also revealed that the combination of financial issues, such as a low level of financial knowledge, low income and high debt might unfavourably influence the FWB of both individuals and households.

The FWB of young adults influences not only their psychological wellbeing but also their life satisfaction, academic success and job performance (Shim et al., 2009; Pandey et al., 2020). In the long run, poor financial situations of adults while they were young may have a detrimental impact on their life wellbeing, interpersonal and familial relationships and their prospects of making a successful transition into the next stage of life (Shim et al., 2009). Meanwhile, socio-economic determinants are critical for FWB (Prawitz and Cohart, 2016; Brüggen et al., 2017). Furnham and Cheng (2017) found in their study that gender, social class, intelligence level, LOC, educational level and occupational status play a significant role in affecting the FWB of young adults.

A survey by Pandey et al. (2020) also found that a substantial percentage of young adults was experiencing low FWB, had difficulty controlling their monthly spending, lacked financial skills and were unsatisfied with their financial situations. However, those who had a conversation and sought financial advice from others about prudent spending behaviour, saving skills, budgeting and investing. On the other hand, they were more likely to have a positive attitude towards money and better financial behaviour in terms of saving, spending and tracking expenses, resulting in improved expected FWB (Pandey et al., 2020). Furthermore, young adults who grew up with healthy financial habits patterned by their parents and elders will have better financial behaviour and FWB and are more likely to attain their financial goals (Ullah and Yusheng, 2020). Individuals who constantly discuss and observe their parents’ financial decision-making process indirectly will strengthen their own FWB.

### FWB and Financial Behaviour of Low-Income Young Adults

Numerous studies have also examined FWB and financial behaviour from the perspective of young or emerging adults. Many of these studies used university students as their sample (e.g., Shim et al., 2009; Falahati et al., 2012; Lanz et al., 2020), although some others used young working adults within a certain age range as respondents (e.g., Arifin et al., 2018; Topa et al., 2018; Yong et al., 2018). The focus on youths and young adults have continued to spur research interest among scholars since this group of the population plays an important role in society, being future leaders that would lead the world into even more challenging circumstances.

Young adults face numerous challenges in their lives and are in the process of learning numerous life skills while transitioning into adulthood. Among the important life skills attained are those related to working and money matters. The literature shows that income is an important determinant of financial health, perceived FWB and financial behaviour (e.g., Cummins, 2000; Bonke and Browning, 2009; Mokhtar and Rahim, 2016; Mahdzan et al., 2019), and this is evident in the findings of numerous studies across different contexts, for example in America (Malone et al., 2010; Netemeyer et al., 2018), China (Chen and Lemieux, 2016; Chen and Jin, 2017), Malaysia (Mokhtar and Rahim, 2016; Mahdzan et al., 2019) and Australia (Buchler et al., 2009). Socio-economic factors, such as education level, are also correlated with financial behaviour and perceived FWB of individuals (Shusha, 2016; Mokhtar and Husniyah, 2017; Mahdzan et al., 2019; Zaimah, 2019; Chauhan and Dhami, 2021). Previous findings have shown that educational level and monthly income are determinants of financial behaviour and FWB (Shusha, 2016), explained that people with better income and higher educational levels exhibit better financial behaviour and higher perceived FWB. From the literature, it was found that researchers have focused on financial aspects of vulnerable groups of society particularly the low-income households (e.g., Loibl, 2017; Hung, 2020; Magli et al., 2021). However, there appears to be very scant number of studies that address a specific segment of the vulnerable group, i.e., the young adults of low-income households. Hence, studies that focus on the financial aspects of low-income young adults are viewed to be worthy. Thus, this is the gap that the present study aims to fill.

### Financial Knowledge and FWB

Financial knowledge is the basic understanding of an individual in subject matters related to finances (Khan et al., 2017; Saurabh and Nandan, 2018). Financial knowledge also refers to the capacity to understand, manage and make decisions related to finance. It describes how someone earns, handles and invests in money, and how they contribute to helping others. More specifically, it refers to the set of skills and knowledge that enables people to make informed and effective financial decisions with all of their resources (Hafizudin et al., 2018).

All individuals should have financial knowledge to financially function well in their daily routines. They should at least be aware of their micro and macroeconomic environment and grasp the basic financial concepts such as savings, investment, credit, interest rates, inflation and consumer goods pricing, to list a few. There are two components of financial knowledge: objective financial knowledge and subjective financial knowledge. Objective financial knowledge refers to the actual financial knowledge of an individual, whereas subjective financial knowledge refers to the confidence level of an individual in making financial decisions (Alba and Hutchinson, 2000; Wang, 2009). According to Khan et al. (2017), financial knowledge is sometimes used interchangeably with financial literacy in this research area.

Previous studies have found that people with a high level of financial knowledge will have better FWB (Falahati and Paim, 2011; Saurabh and Nandan, 2018; Sabri et al., 2020a). Financial knowledge can benefit a person by providing them with a wide range of skills and insights. For instance, skills that help them manage their finances, better insights into financial concepts and familiarity with financial institutions (Albeerdy and Gharleghi, 2015), which at the same time may increase their FWB. Previous studies also argued that individuals with a higher level of financial knowledge are better at managing their finances, which leads to better financial behaviour and FWB (Hilgert et al., 2003; Britt et al., 2013). Shim et al. (2009) agreed that one of the right ways to establish and maintain the FWB of people is by increasing their financial knowledge. The Financial Behaviour Survey conducted by the Credit Counselling and Debt Management Agency (2018) has also shown a positive association between the degree of financial knowledge and FWB, further affirming the significance of securing financial knowledge among individuals. Therefore, based on past literature, this study hypothesises the following:

*Ha1*: Financial knowledge positively correlates with the perceived FWB of low-income young adults.

### Locus of Control and FWB

According to Rotter (1966), LOC is the control of individuals over their work and their beliefs about self-success. Britt et al. (2013) defined LOC as the degree to which individuals believe they are in charge of their future. There are two dimensions of LOC: internal and external. Individuals with an internal LOC feel that their actions determine future occurrences and within their control, whereas those with an external LOC believe that future events are determined by luck, chance, fate and out of their control (Jorgensen et al., 2017). Concerning the financial LOC, people with a high internal LOC generally believe that their financial gains or problems are consequences of their efforts and hard work. In contrast, those with a high external LOC generally believe that their success or failure in finance happened because of external factors beyond their control, such as bias, injustice, fate, luck, circumstance or the faults of others. Rotter (1966) also explained that people with a strong internal LOC are more likely to: (i) be aware of their surroundings, (ii) make efforts to improve their surroundings, (iii) value their skills and be concerned about their abilities and (iv) be resistant to subtle attempts to influence them.

Even though LOC is not a new concept, the number of studies on the financial LOC is limited (Kesavayuth et al., 2018). According to past literature, LOC is one of the most commonly studied personality traits in personality and applied psychology (Judge and Bono, 2001). However, social scientists have only begun to substantially link LOC with financial concepts in recent years (Ullah and Yusheng, 2020). As a result, only some prominent researchers have looked into the relationship between LOC and financial concepts, for instance, FWB. Previous studies also found that LOC influences individuals’ financial or non-financial behaviours of individuals (Kesavayuth et al., 2018). Rather than corporate finance, Jorgensen et al. (2017), on the other hand, agreed that internal LOC is more relevant in the studies of personal finance.

Individuals with an internal LOC were more likely to experience FWB (Prawitz and Cohart, 2016). Moreover, Furnham and Cheng (2017) also agreed that LOC is one of the indicators associated with FWB, together with parental social class, education level, childhood intelligence measures and self-esteem. Individuals with an internal LOC are more likely to work hard to achieve their goals. Thus, the FWB of individuals will be higher for those who believe in their own, observe the financial behaviours of others and discuss financial-related subjects with their significant others (Ullah and Yusheng, 2020). Hence, the present study hypothesised the following:

*Ha2*: Internal LOC positively correlates with the perceived FWB of low-income young adults.

### Mediating Role of Financial Behaviour

Gudmunson and Danes (2011) defined financial behaviour as a ‘pattern of actions over time such as earning, saving, spending, and gifting’. Financial behaviour is any human activity that has something to do with money management or habits. Cash, credit and savings are all common financial behaviours. Jorgensen et al. (2017) explained financial behaviour in the cash management, credit management, capital accumulation and general management domains. A previous study explained that healthy financial behaviour reflects some positive habits, such as budgeting, paying bills on time, making a spending plan, cash flow management, credit management and retirement planning (Kapoor et al., 2004). In contrast, lack of budget planning and excessive debt accumulation are examples of poor financial behaviour, which leads to financial distress and financial difficulties (Malaysian Financial Planning Council, 2016).

Past literature also found that most young adults have problems managing money and planning their expenses to satisfy their day-to-day financial commitments, needs and wants. A survey conducted by Mien and Thao (2015) revealed that most young adults spent the most on apparel, cosmetics, movie tickets and dining in fancy restaurants to show off their lifestyle and to impress others. With current social media trends such as Instagram, TikTok and many more, these habits could be worse than before. These poor financial behaviours and habits significantly impact their personal lives.

Financial knowledge may encourage positive financial behaviours (Lajuni et al., 2018; Baptista and Dewi, 2021; Chong et al., 2021), such as spending only on necessities (Ibrahim et al., 2010; Farinella et al., 2017), paying bills on time, increasing savings and investing, managing credit cards wisely (Lusardi et al., 2010; Laily, 2014), having a proper retirement plan (Klapper and Panos, 2011) and getting personal and family health insurance coverage (Kim et al., 2013; Bauhoff et al., 2020). On the other hand, negative financial behaviours are regularly causing financial issues, with poor financial knowledge being a significant cause (Lusardi and Tufano, 2015; Sabri et al., 2020a). Social scientists believe that higher skills and knowledge in managing finance will enable young adults to practice better financial behaviour in their daily lives.

It was also hypothesised that LOC would influence the financial behaviour of young adults. Previous studies indicated that college students with an external LOC exhibit the worst financial behaviours (Britt et al., 2013; Mien and Thao, 2015). According to Britt et al. (2013), college students with a high external LOC were more likely to report a lower degree of financial behaviour. In contrast, young adults with a better internal LOC, who thought they had more control over the outcomes of their lives, acted more responsibly in financial issues (Jorgensen et al., 2017). In other words, most social scientists believe that while a high internal LOC is associated with positive financial behaviour, a high external LOC may cause the opposite.

This study investigates the role of financial behaviour as a mediator of the associations between financial knowledge, LOC and perceived FWB among young adults with low incomes controlling for education and income effects. Previous studies have argued that financial knowledge and LOC are factors that improve financial behaviour (Britt et al., 2013; Jorgensen et al., 2017). These factors affect FWB (Falahati and Paim, 2011; Sabri et al., 2020b). Saurabh and Nandan (2018) also agreed with the associations between these variables. Low financial knowledge may lead to financial behaviour issues (Idris et al., 2013), such as income management, retirement planning, savings and contingency planning of individuals, eventually affecting their FWB (Sabri et al., 2020a). Previous research has also shown that positive financial behaviour is related to better FWB (Joo and Grable, 2004; Chong et al., 2021). In a family resource management model with an input-throughput-output mechanism, the impact of inputs on outputs is typically indirect (Beutler and Sahlberg, 1980). Thus, this study examined the function of financial behaviour as a mediator variable in the associations between independent and dependent variables. This study hypothesises that as:

*Ha3*: Financial knowledge positively influences the financial behaviour of young low-income adults.

*Ha4*: Internal LOC positively influences the financial behaviour of low-income young adults.

*Ha5*: Financial behaviour positively influences the perceived FWB of low-income young adults.

*Ha6*: Financial behaviour mediates the association between financial knowledge and perceived FWB of low-income young adults.

*Ha7*: Financial behaviour mediates the association between the internal LOC and the perceived FWB of low-income young adults.

## Research Methodology

### Data and Sample

Multi-stage sampling was used to select the sample in the present study. In the first stage, using the random sampling technique, four states were selected from four different zones (i.e., central, southern, northern and eastern) in Peninsular Malaysia and two states from East Malaysia. In the second stage, the information of respondents was identified *via* the list of the National Household Sampling Frame (NHSF) and consultation support from the Malaysian Department of Statistics. The research criteria of the respondents in this survey were young adults, aged between 18 and 35 years old, resided in urban areas and earned less than RM4,850 per month, which classifying them as a low-income group. Once again, using the random sampling technique, the samples were selected based on the enumeration block (EB) and residential places (RP). A total of 535 responses from the six states (that is, four zones in Peninsular Malaysia and two states from East Malaysia) were obtained from the distributed questionnaire. After removing responses with missing values and straight-lining issues, 482 usable data were used in the present study. Next, CB-SEM was further being utilised as an analysis tool to test the mediation effect by financial behaviour for the influence of exogenous construct on perceived FWB as an endogenous construct.

There are many disputes in the literature about the optimal number of sample sizes to acquire in order to use CB-SEM. Gorsuch (1983) and Kline (1994) recommended a sample size of 100 as the absolute minimum, but Comrey and Lee (1992) recommended sample sizes of 100 as inadequate, 200 as acceptable, 300 as good and maximum of 500 sample as very good sample size required (Mundfrom et al., 2005; Awang, 2015; Hair et al., 2017, 2019). However, if the size of the data set is too small, CB-SEM might yield aberrant findings when the data are not normal (Hair et al., 2019). Moreover, CB-SEM performance is significantly improved in the structural model, where relative deviations for small sample sizes are equivalent to those obtained by factor-based SEM for sample sizes of 250–500 (Reinartz et al., 2009). In addition, scholars have highlighted that CB-SEM maximum likelihood estimate is resilient to breaches of normality (Chou et al., 1991; Olsson et al., 2000). As a result, the sample size of 482 employed in this study is sufficient for CB-SEM analysis.

### Data Collection

A cross-sectional survey design was employed to collect data and meet the objective of this study. Before the actual data collection process, a series of pilot and pre-tests were conducted to ensure the validity and reliability of the questionnaire. First, a pre-test was tested on the suitability and consistency of the questionnaires, together with the support of five academicians and industrial experts. At this stage, the contents of the instruments used in the questionnaire were validated by the invited experts. In the second stage, in-depth interviews were conducted with 15 prospective respondents to identify potential issues related to the questionnaire, such as format, designs, wording and instrument clarity. The estimated time duration was tested to answer a complete set of questionnaires. Based on the feedback from the in-depth interview session, researchers made minor corrections regarding the questionnaire layout and wording. In the third stage, a pilot test was conducted among 50 respondents from low-income households to represent the respondents in the present study. Based on the results obtained during the pilot test, the last minor amendments were performed. The application for ethical clearance (approval) for this study has been approved by the Ethics Committee for Research Involving Human Subjects, Universiti Putra Malaysia. The reference number is JKEUPM-2020-171.

During the actual data collection stage, the respondents were briefed by enumerators before they answered the questionnaire. The purposes and rationale of the research, their voluntary involvement in the study and monetary incentives were covered in the briefing. On a voluntary basis, no cash incentives were offered to respondents. The sampling method and approach were used because of the time and economic limitations of the study. Moreover, this approach does not compromise the performance of reliable inferences from statistical analysis.

The unit of analysis for this study consisted of young adults aged between 18 and 35 years old. The selection of young adults was relevant because they represent a large portion of the Malaysian population. According to the Malaysian Population Piramid, reported by the World Population Review (2022), approximately 31.2 percent of the total population in Malaysia are young adults, representing 43.9 percent of the adult population. These numbers were supported by the Population Distribution and Basic Demographic Characteristic Report published by the Department of Statistic Malaysia (2010). Statistics also show that a large number of young adults in Malaysia have declared bankruptcy. Based on a statistic report from the Malaysian Insolvency Department, 25.3 percent of bankruptcy cases from 2016 to 2020 consist of young people aged below 35 years (Bankruptcy Statistic, 2020). These data indirectly suggest that this group of samples is particularly needed in terms of perceived FWB.

### Instrument and Measurement of Variables

Self-administered questionnaires were used in the present study. The questionnaire consisted of five sections, starting with the demographic and socio-economic profile, followed by questions related to financial knowledge, LOC, financial behaviour and perceived FWB.

#### Financial Knowledge

The financial knowledge level of respondents was measured using 10 true and false questions, in which a higher number of questions answered correctly, equal to a better level of financial knowledge. These questions were adapted from the Malaysian-based scale developed by Sabri et al. (2006). The scale consisted of statements related to the subtopics of general financial knowledge, savings, investment, credit cards, debts, loans, Islamic banking and products. This measurement has been adopted and validated in previous Malaysia-based financial studies (Sabri and Aw, 2019, 2020). Both the alterations in the financial landscape and the Islamic banking introduction have been created new dimension and extraordinary banking sector in Malaysia. As a result of this circumstance, customer preferences and demands for better and high-quality banking services have also been shifted. Moreover, a vast array of banking products and services has been offered according to their preferences for both Muslim and non-Muslim customers through the establishment of more financial institutions both conventional and Islamic in recent years (Abdullah et al., 2012). A significant quantity of literature has recently emerged on the topic of financial knowledge or financial literacy. Even though, a resurgence of interest in Islamic finance among academics has also been arisen (Chong and Abdullah, 2014), many of the developed measurement scales were not compatible with the philosophy of the Islamic finance concept. Concurrently, no effort has been proposed yet to provide an adequate multi-country scale, with the exception of Islamic ‘banking-only’ literacy scales. In other words, there is no complete financial knowledge or literacy scale in the literature that includes all aspects of Islamic finance, including banking, insurance, capital markets and any other Sharia-compliant financial model (Dinc et al., 2021). Therefore, Islamic banking was included in the scale to measure the financial knowledge in the current study.

#### Locus of Control

The measuring instrument for LOC was adapted from Sumarwan and Hira (1993). This instrument consists of eight items for internal locus of the control scale. The items in the scale were measured using four Likert scales, ranging from Strongly Disagree (1), Disagree (2), Agree (3) and Strongly Agree (4). A higher total score for the internal subscale reflects a better level of internal LOC. In contrast, a lower total score for the external subscale indicates a better external LOC.

#### Financial Behaviour

Recognising that financial behaviour encompasses various subcategories of personal financial management, this study incorporates the aspects of managing expenditures, saving, managing loans and long-term financial goals to provide a comprehensive measurement of financial behaviour. The measurement scale of financial behaviour was adapted from a nationwide study by the Malaysian Financial Planning Council (2016), designed to suit the Malaysian context. This scale was measured using a four-point frequency scale ranging from Never (1) to Always (4) and consists of eight positive and two negative items. A higher total score reflects a higher level of positive financial behaviour. This measurement has also been adopted and validated in previous Malaysia-based finance studies (Sabri and Aw, 2019, 2020).

#### Perceived FWB

The perceived FWB of young adults in this study was measured using eight questions adapted from a Malaysian context questionnaire developed by Garman and Jariah in 2006 (Jariah, 2007), also known as the Malaysian Financial Wellbeing Scale (MFWBS). This scale investigated the respondents’ concerns about general satisfaction with their financial situation, ability to meet daily expenses, financial management, financial sufficiency and current financial satisfaction. It consists of eight positive items, measured using four Likert scales, ranging from Strongly Disagree (1) to Strongly Agree (4). A higher total score reflects a better level of perceived FWB of respondents.

### Conceptual Model

**Figure 1:**
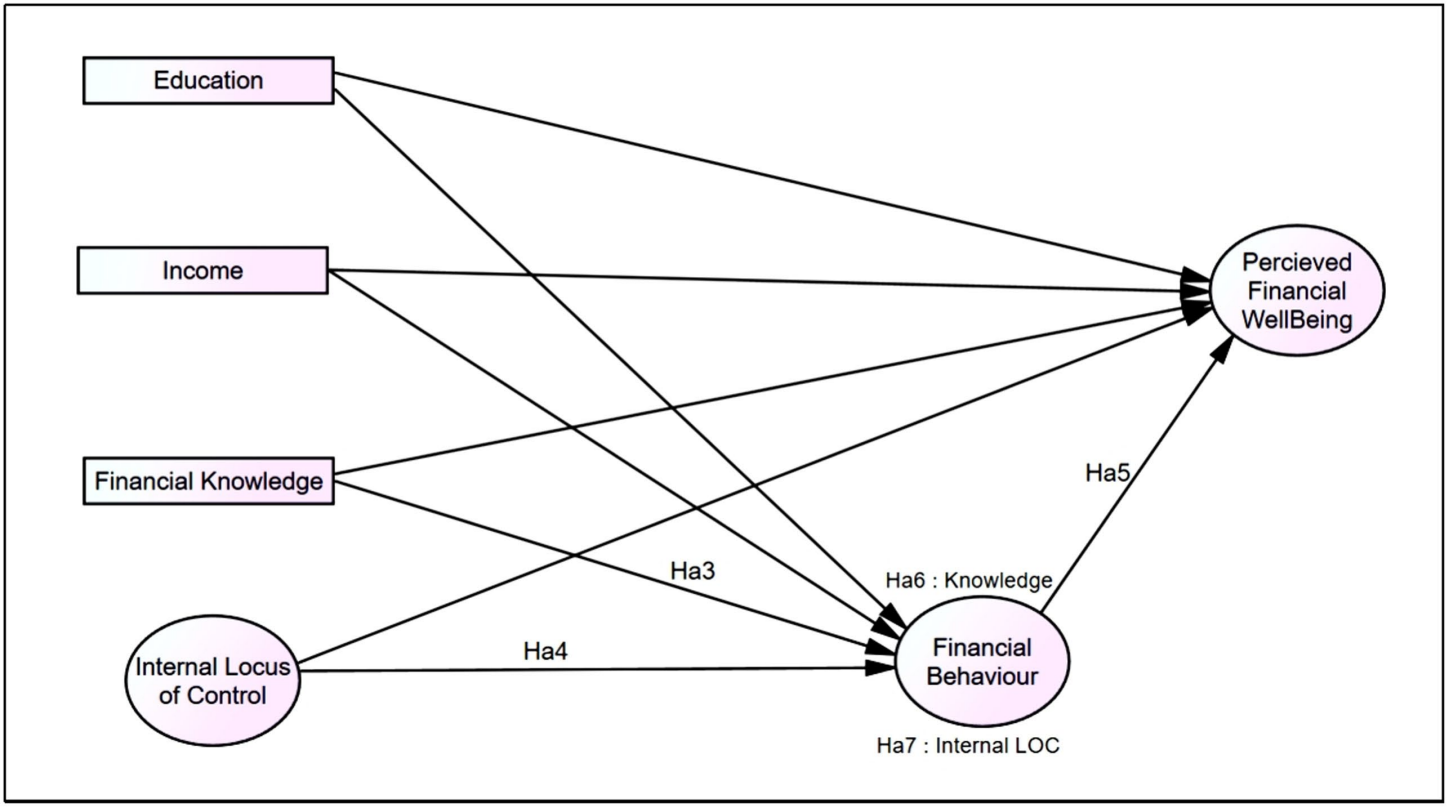
Conceptual framework.

In this study, a conceptual model was proposed to examine perceived FWB among low-income young adults and examine the efficiency with which this model describes the relationships among four constructs: (i) financial knowledge, (ii) internal LOC, (iii) financial behaviour and (iv) perceived FWB (see Figure 1) controlling for education and income. Relationships among independent constructs and FWB as a dependent construct are determined using Pearson correlation in SPSS version 27.0 for hypothesis Ha1 and Ha2. IBM SEM AMOS 28.0 is being utilised as an analysis tool to test the mediation effect by financial behaviour for the influence of exogenous construct on perceived FWB as an endogenous construct. As can be seen from Figure 1, researchers test the direct and indirect effect of the exogenous construct (financial knowledge, internal LOC) on perceived FWB. Financial behaviour acts as the mediator in the FWB model.

## Findings and Discussion

### Data Analysis

The overall data analysis process consisted of five steps. Firstly, the researchers calculated descriptive statistics to identify the univariate characteristics for socio-economic construct. Secondly, the researchers computed Cronbach’s alphas to demonstrate the internal consistency of every discussed construct. Then, the researchers investigated the bivariate relationships between all the studied constructs using the Pearson correlation test. Subsequently, the researchers tested the model fitness of the perceived FWB and mediation model using a CB-SEM analysis.

### Profile of Respondents

This study was conducted among Malaysian young adults aged between 18 and 35 years old. From these total numbers, approximately 2.4 percent of the respondents belonged to the age category below 20 years old, 60.9 percent were in their 20s, aged between 20 and 29 years old and 36.7 percent were in their early 30s, aged between 30 and 35 years old. About 63.3 percent of the respondents were male, while 36.7 percent were female. The majority of the respondents was Malays (72.6%), followed by Sabah native (14.9%), Indians (6.2%), Sarawak native (3.1%), Chinese (2.7%) and other ethnicities (0.4%). More than half of the respondents (54.6%) were married, 41.3 percent were single, 3.3 percent were divorced and 0.8 percent were separated from partners. In terms of education level, more than half of the respondents (61.0%) had a low education (primary and secondary) level, and 39.0 percent had a tertiary level of education. Among young adults who had tertiary level of education, 19.9 percent of the respondents had diplomas and 19.1 percent had bachelor and master degrees.

### Reliability Tests

Reliability coefficients of the instruments used in this study were identified based on Cronbach’s alpha values from the ABM SPSS. The reliability coefficients for the five constructs ranged from Cronbach’s alpha 0.75 to 0.88, satisfying the criterion of exceeding 0.7 (Nunnally, 1967). As for the details, Cronbach’s alpha value for financial knowledge is 0.75, Cronbach’s alpha values for constructs internal and external LOC are 0.78 and 0.82, respectively. The value of Cronbach’s alpha for financial behaviour was 0.81, while Cronbach’s alpha for perceived FWB was 0.88.

### Bivariate Correlation Analysis

Table 1 shows the coefficient of correlation among the constructs in the present study. Generally, all of the exogenous construct in the present study were significantly correlated with perceived FWB, all in the positive direction. The magnitudes of all correlations ranged from 0.128 to 0.398.

**Table 1:** Coefficient of correlations of variables.

	Financial knowledge	Internal LOC	Financial behaviour
Financial knowledge			
Internal LOC	0.117^*^ ^*^		
Financial behaviour	0.115^*^ ^*^	0.358^*^ ^*^	
FWB	0.128^*^ ^*^	0.291^*^ ^*^	0.398^*^ ^*^

*SD, standard deviation*.

** *Significant at the 0.001 level (two-tailed)*.

In particular, based on Pearson correlation tests, financial knowledge (*r* = 0.128, *p* < 0.01), internal LOC (*r* = 0.291, *p* < 0.001) and financial behaviour (*r* = 0.398, *p* < 0.001) were positively and significantly correlated with perceived FWB. The results also showed that financial knowledge (*r* = 0.115, *p* < 0.01) and internal LOC (*r* = 0.358, *p* < 0.001) were significantly correlated with financial behaviour. As such, hypotheses 1, 2, 3, 4 and 5 were supported. On the other hand, financial knowledge was also significantly related to internal LOC (*r* = 0.117, *p* < 0.01) in the positive directions.

Pearson correlation tests revealed that low-income young adults who have better financial knowledge or higher internal LOC seem to have a better level of perceived FWB. In addition, they seem to have a better level of financial behaviour. This study also found that financial behaviour positively influenced the level of perceived FWB of low-income young adults in Malaysia. This finding clarifies that young adults who have better financial behaviour would have greater perceived FWB.

The results of this study are also consistent with those of previous studies, which indicated that financial knowledge affects FWB (Falahati and Paim, 2011; Saurabh and Nandan, 2018; Radianto et al., 2021) and financial behaviour (Lajuni et al., 2018; Sabri et al., 2020a; Baptista and Dewi, 2021) of the individuals. Young adults have many financial responsibilities and have just started their financial journey independently. Thus, the foundation of financial knowledge is critical. It may impact not only their wellbeing financially but also other aspects of life. Therefore, individuals need to seek financial knowledge starting at an early age. Financial knowledge can be an early preparation for individuals before they enter the adult stage.

The present study provided evidence that low-income young adults with a higher internal LOC would report better perceived FWB. Prawitz et al. (2013) also agreed in their study that even in the face of severe economic hardship, those with a better internal LOC orientation reported greater FWB. This finding is also in line with previous studies (Prawitz and Cohart, 2016; Jorgensen et al., 2017; Ullah and Yusheng, 2020). Most social scientists have reported significant relationships between the internal LOC with perceived FWB. Young adults with a better internal LOC believe that they are in control of their fates. Thus, they work on themselves to improve their lives.

### Model Fit

Influential factors for perceived FWB were determined using the Structural Equation Modelling, in which perceived FWB was the outcome construct. The model comprised financial behaviour, financial knowledge and internal LOC as predictor construct with financial behaviour as a mediator. Socio-economic constructs were used such as education and income as control constructs. The fitness of the perceived FWB model was analysed using fitness indexes as shown in Table 2. The results indicated that the absolute fit of RMSEA was 0.038 while incremental fit comprising of CFI achieved 0.971, TLI 0.963 and NFI 0.935. Parsomonious fit presented by Chisq/df displayed an index value of 1.708. Thus, these concluded that all the index categories achieved the requirement of a fit model.

**Table 2:** Index category and the level of acceptance.

Category	Name of index	Index value	Result
1. Absolute fit	RMSEA	0.038	Achieved the requirement
2. Incremental fit	CFI	0.972	Achieved the requirement
	TLI	0.962	Achieved the requirement
	NFI	0.936	Achieved the requirement
3. Parsimonious fit	Chisq/df	1.731	Achieved the requirement

R square value for this mediation model was 0.34, which indicated that the model explained 34.0 percent of the variance in the perceived FWB. This model was a good fit model as supported by Cohen (1992) stating that 26 percent and above represent a high effect size. This finding showed that the exogenous variables together explained 19.7 percent of the variance in perceived FWB among low-income young adults in the present study, with financial behaviour serving as the most important predictor.

A high factor loading for the measuring items in each construct of higher than 0.5 (Maskey et al., 2018) indicates unidimensionality is achieved. Items having factor loading below 0.4 were deleted. Furthermore, the correlation between exogenous construct ranges from −0.1 to 0.35 also demonstrate unidimensionality of the model.

A low correlation between exogenous construct indicates a weak correlation and the discriminant validity is achieved (Table 3). The construct reliability value of at least 0.6 showed that the model achieved construct reliability (C.R. = 0.840 for perceived FWB; 0.818 for internal LOC; and 0.696 for financial behaviour). Average variance extracted (AVE) displaying a value of at least 0.5 is required for a fit model. The values achieved in this model were 0.5 for perceived FWB; 0.6 for internal LOC; and 0.4 for financial behaviour. A value of 0.4 for AVE can be accepted as mentioned by Fornell and Larcker (1981) if the composite reliability is higher than 0.6.

**Table 3:** Convergent validity and reliability for the constructs.

Construct	Item	Factor loading	CR	AVE
Financial wellbeing	V_H_8	0.63	0.840	0.50
	V_H_5	0.73		
	V_H_4	0.83		
	V_H_2	0.71		
	V_H_3	0.59		
	V_H_7	0.59		
Internal locus of control	V_G_6	0.75	0.818	0.60
	V_G_5	0.86		
	V_G_4	0.71		
Financial behaviour	V_E_3	0.55	0.696	0.40
	V_E_5	0.65		
	V_E_7	0.73		
	V_E_10	0.47		

### Mediation Analysis

**Figure 2:**
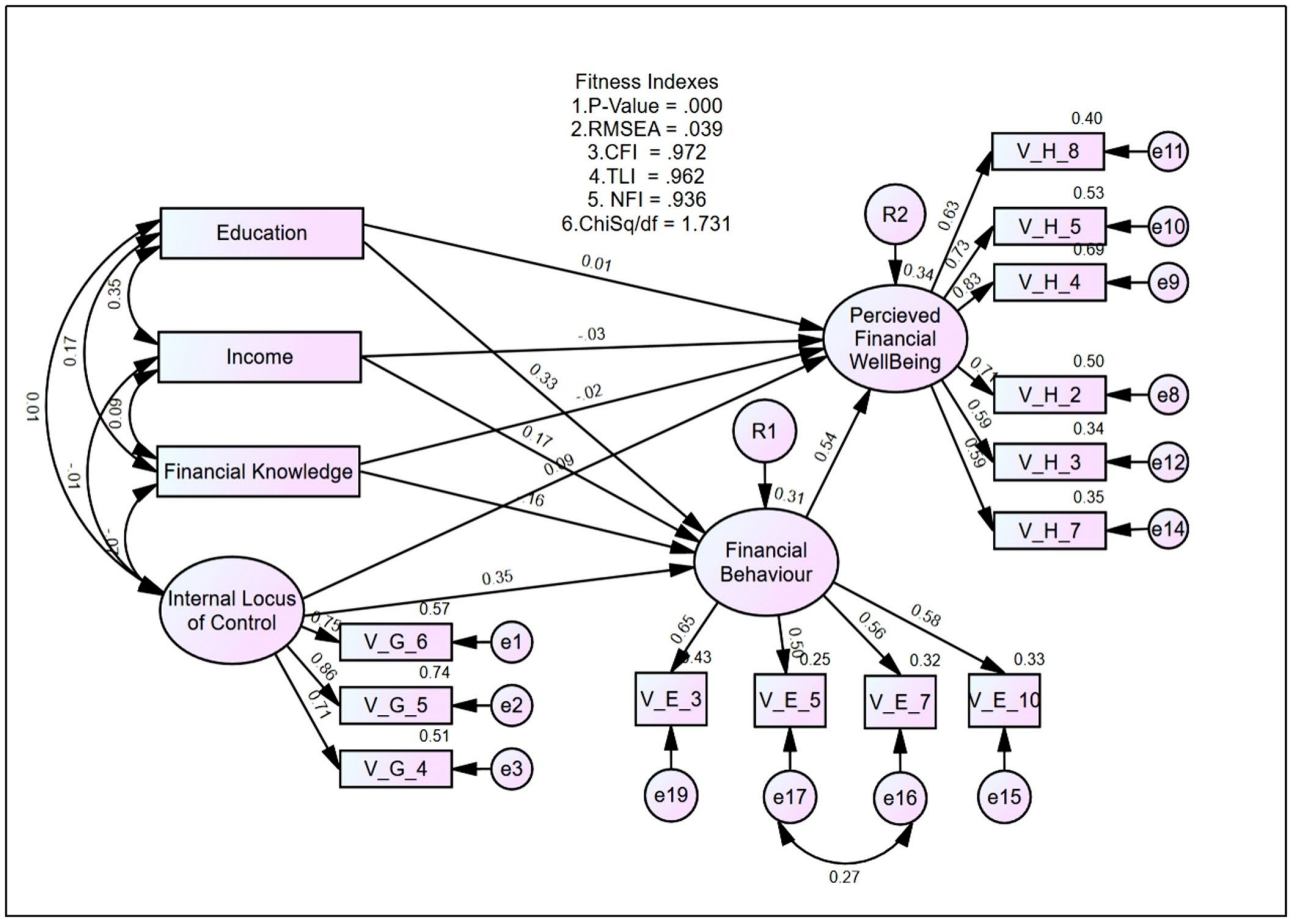
Structural equation modelling of perceived FWB.

The mediation hypotheses in the present study were tested using CB-SEM. Figure 2 shows the mediation model illustrating the influence by exogenous constructs on the endogenous construct that may be indirect or direct. The direct effect happens when the relationship between X on Y cannot be influenced by a third variable. The indirect effect occurs when the relationship between X and Y is influenced (or mediated) by another variable(s). The estimate of the relationship between X and Y gives the direct effect. Indirect effect is calculated from the multiplication of the estimates for the X mediator and mediator Y relationships.

Table 4 displays the results for the mediation model in this study. For hypothesis Ha3: Financial knowledge positively influences the financial behaviour of young low-income adults, the table displays a significant influence (Estimate = −0.050; *p*-value = 0.001) and Ha3 is supported. Thus, the regression weight for financial knowledge in the prediction of financial behaviour is significant at the 0.001 level. A knowledgeable young adult in finances would tend to display a positive financial behaviour. This result is concurrent with previous studies (Hilgert et al., 2003; Britt et al., 2013; Credit Counselling and Debt Management Agency, 2018).

**Table 4:** Direct and indirect effect for the mediation model of perceived FWB.

			Estimate	SE	C.R.	*p*
Financial behaviour	<---	Education	0.049	0.009	5.678	***
Financial behaviour	<---	Income	0.000	0.000	3.016	0.003
Financial behaviour	<---	Financial_Knowledge	−0.050	0.016	−3.182	0.001
Financial behaviour	<---	Internal Locus of Control	0.331	0.055	6.065	***
Perceived financial wellbeing	<---	Financial Behaviour	0.779	0.110	7.079	***
Perceived financial wellbeing	<---	Education	−0.005	0.010	−0.479	0.632
Perceived financial wellbeing	<---	Income	0.000	0.000	−0.679	0.497
Perceived financial wellbeing	<---	Financial_Knowledge	−0.008	0.019	−0.398	0.691
Perceived financial wellbeing	<---	Internal Locus of Control	0.099	0.067	1.479	0.139
Perceived financial wellbeing	<---	Income	0.000	0.000	−0.679	0.497

*** *Significantly different from zero at the 0.001 level (two tailed)*.

Internal LOC exhibits a positive influence on the financial behaviour of low-income young adults. Thus, Ha4 of this influence is supported (Estimate = 0.331; *p*-value = 0.001). Those young adults having high internal LOC would be able to demonstrate a positive financial behaviour (Jorgensen et al., 2017; Kesavayuth et al., 2018; Ullah and Yusheng, 2020). The ability to control oneself is important in controlling their activities regarding finances.

The fifth hypothesis (Ha5) on the effect of financial behaviour on perceived FWB stating that financial behaviour positively influences the perceived FWB of low-income young adults is also supported by the result (Estimate = 0.779; *p*-value = 0.001). Practicing good financial behaviour would lead to a better financial wellbeing among young adults (Husniyah et al., 2017; Magli et al., 2021). Making good decisions in financial matter and choosing the financial products to elevate ones financial wellbeing has proved to be a contributing factor.

In testing the hypothesis on mediation effect of financial behaviour, Ha6: Financial behaviour mediates the association between financial knowledge and perceived FWB of low-income young adults, a full mediation result is revealed. The significant indirect effect of financial knowledge on perceived FWB is higher (−0.050 × 0.779 = −0.0390) than the direct effect of −0.008. It is a full mediation since the direct effect of financial knowledge on perceived financial wellbeing is not significant (Estimate = −0.008; *p*-value = 0.691). This mediation result by financial behaviour is consistent with past studies (Saurabh and Nandan, 2018; Setiyani and Solichatun, 2019). With the significant indirect effect and insignificant direct effect, hypothesis 6 was supported. This analysis results proved that financial behaviour was a mediating construct in the influence of financial knowledge on perceived FWB. Thus, it also explains that the financial knowledge of young adults in Malaysia associated with their perceived FWB indirectly through its influence on financial behaviour.

Finally, the seventh hypothesis (Ha7) with regard to the mediating effect of financial behaviour in the influence of internal LOC on the perceived FWB of low-income young adults also exhibits a full mediation result. The significant indirect effect of internal LOC on perceived FWB is higher (0.339 × 0.779 = 0.264) than the direct effect of 0.099. A full mediation is the result since the direct effect of internal LOC on perceived financial wellbeing is not significant (Estimate = 0.099; *p*-value = 0.139). This mediation result by financial behaviour is consistent with scarce past studies (Iramani and Lutfi, 2021; She et al., 2022). This finding explains that the internal LOC of low-income young adults in Malaysia was indirectly associated with their perceived FWB through its influence on financial behaviour.

The present study believed that most low-income young adults in Malaysia faced some problems in achieving perceived FWB due to the lack of financial knowledge and poor financial behaviour. Most social scientists acknowledge that financial knowledge plays a crucial role in improving financial behaviour, which leads to better perceived FWB. The present finding is also in line with a survey conducted among working adults in Malaysia (Credit Counselling and Debt Management Agency, 2018) and other past literature (Falahati et al., 2012; Jorgensen et al., 2017; Saurabh and Nandan, 2018; Setiyani and Solichatun, 2019). In addition, a local study by Sabri et al. (2020a) also supported that mismanagement of income and financial resources caused an increase in the number of young adults going bankrupt in Malaysia, which has a detrimental impact not only on individuals but also on their families and society. In addition to financial knowledge, this study also found that both internal and external LOC were indirectly associated with the perceived FWB of Malaysian low-income young adults through their financial behaviour. This finding is in line with some other notable studies (Perry and Morris, 2005; Grable et al., 2015; Ullah and Yusheng, 2020; Radianto et al., 2021), which also acknowledged the association between LOC, financial behaviour and FWB.

## Conclusion and Implications

The present study develops and tests a conceptual model of perceived FWB based on financial behaviour, financial knowledge and LOC having education and income as control variables. This study is among the few pioneering studies that explore the link between financial knowledge, LOC, financial behaviour and perceived FWB among low-income young adults in Peninsular and East Malaysia. The findings from this study confirm the indirect effects of financial knowledge and internal LOC on perceived FWB, through financial behaviour among low-income young adults in Malaysia. In addition, the results proved that financial behaviour is the strongest predictor of perceived FWB, followed by LOC.

This study also found an interesting fact that, although not as the main findings, it is noteworthy. The majority of the young adults in this study are still a part of the low-income group, despite having a higher level of education because of the low minimum monthly wage policy in Malaysia. The minimum monthly wage is RM1,200 (USD 286) and it may be a total of RM2,400 (USD572) for a household which falls in the B40 group with a border of RM4,850 (USD 1,156). Moreover, this group of young adults are those earning income within the B40 group during the current pandemic situation. Due to the MCO, some have been retrenched or out of business pushing them out from the M40 group and laid in the B40 group. The economic situation during the COVID-19 era limited the job offers and unemployment rate rises from 3.3 percent in 2019 to 4.5 percent in 2020 and further up to 4.6 percent in 2021 (Department of Statistic Malaysia, 2022).

Thus, the link between low income, high cost of living and perceived FWB is worth discussing and needs to be considered by policy makers and Malaysian leaders. These issues should be investigated in future studies in Malaysia. Furthermore, given the current economic recession in recent years and pandemic situation, as well as the high standard of living, high prevalence of debt and financial management issues confronting the younger generation, the authors examined this aspect of young adult development and sought to understand how perceived FWB may be related to behaviours and personal values such as LOC. As a result, the authors developed an interdisciplinary model that combines psychological and behavioural perspectives to explore the role of perceived FWB in the lives of young adults.

Poor financial practices and a lack of financial education affect a large number of young adults in Malaysia. Therefore, the present study suggests that financial knowledge gained through formal and informal education systems is crucial for individuals to help them develop good habits or behaviours in financial and lead to better perceived FWB. Schools and colleges should introduce the subject of financial education as part of their curriculum. However, achieving positive perceived FWB is a complex process that cannot be achieved only through education. It is affected by other socialisation aspects, and young people themselves must play a vital role in this matter. Without the other crucial components, financial knowledge alone is unlikely to contribute to the absolute impact on perceived FWB or life success.

The process of increasing financial ability should also start at an early age, with the bits of help from all the closest elements in their system, such as parents, teachers or significant others. Parents and family members play a crucial role in shaping good financial behaviours and habits among the younger generation by setting outstanding examples. Families and related local institutions must also encourage young people to save and educate them not to be impulsive with unplanned purchases. Before making any financial decisions, they need guidance to evaluate alternative financial products, policies or companies involved. In addition, young adults must know the value of good financial habits such as budgeting, spending constraints, continual expense monitoring, saving and planning for later life and unforeseen needs, and most importantly, living within their means. The authorities must also ensure that adolescents and young adults have easy access to financial experts and counsellors. This effort may help them reach professional advice instead of invalid information from the Internet or other unknown resources.

Based on the findings, this study also recommends implementing ongoing awareness initiatives for teachers, parents and children. These programmes should emphasise the necessity of sharing information and practicing good financial habits. Furthermore, these programmes might be incorporated into early childhood education at schools to encourage children to save and manage money wisely from an early age. Rather than theoretical concepts of financial management, content should focus on experience-based and practical learning. As a result, children will learn about money-related activities and enhance their perceived FWB in adulthood. Another crucial factor is that parents, teachers and peers should instil confidence in managing their issues, which will assist them in making better financial decisions and adapting to the changes and crises.

Apart from having good financial practices in elevating financial wellbeing among households, as job offers are limited and employment income may be inadequate to sustain good living, job-seekers may opt to be self-employed harnessing on their entrepreneurial soft-skills and hard skills. Descriptive analysis on their skills revealed business on line (21.0%) followed by cooking (10.4%), grab driver (8.1%), carpentry (6.4%) and baking cakes (5.8%) are among the skills used to increase their income and further contributing to their financial wellbeing. Others that have not acquired these skills have responded to increase their ability in online business (17%), cooking (14.5%) and repairing cars (11.6%) as they for-see the potential of the skills in upgrading their financial situation.

## Limitations and Recommendations

This study has several notable findings and some important implications for individuals, parents, teachers, society and government, but it has two limitations that should be addressed in future research. Firstly, this was a cross-sectional study. By using cross-sectional data, this study could not adequately explore causal relationships among variables. Longitudinal data are required to test the causal connections. In countries where secondary data on household or consumer finances are available, conducting longitudinal studies would provide more insights on long-term perceived FWB of young adults throughout their life cycle. Unfortunately, in Malaysia, there is a lack of nationwide secondary data that focuses on household finances that researchers could employ. Undoubtedly, a causal model would help us to better understand the development of perceived FWB as well as its origins and the socialisation process, the personal and psychological factors and the various pathways one takes into adulthood. Second, this study focused only on young adults of low-income households in Malaysia. Future studies could make comparisons on the determinants of perceived FWB for other age groups besides young adults and/or examine differences across young adults from other household income groups such as those from middle-income and high-income households. A comparative study would provide better understanding on the deficiencies in financial behaviour and other financial attitudes across the different age or household income groups.

## Data Availability Statement

The raw data supporting the conclusions of this article will be made available by the authors, without undue reservation.

## Ethics Statement

The studies involving human participants were reviewed and approved by the Ethic Committee for Research Involving Human Subject, Universiti Putra Malaysia, Serdang, Malaysia. The patients/participants provided their written informed consent to participate in this study.

## Author Contributions

MS is the lead researcher and was responsible for supervising, developing instruments, reviewing and editing manuscript. RW was responsible for running data analysis, writing original draft, reviewing and editing manuscript. NM was responsible for developing instruments, reviewing and editing manuscript. AM and HA were responsible for running data analysis, developing instruments and collecting data. All authors contributed to the article and approved the submitted version.

## Funding

This work was supported by the Ministry of Higher Education Malaysia through the Long Term Research Grant Scheme–Malaysia Research University Network (LRGS-MRUN), project code (LRGS/1/2016/UKM/02/1/4) entitled “Determinants of Financial Well-being among B40 Households”.

## Conflict of Interest

The authors declare that the research was conducted in the absence of any commercial or financial relationships that could be construed as a potential conflict of interest.

## Publisher’s Note

All claims expressed in this article are solely those of the authors and do not necessarily represent those of their affiliated organizations, or those of the publisher, the editors and the reviewers. Any product that may be evaluated in this article, or claim that may be made by its manufacturer, is not guaranteed or endorsed by the publisher.
